# Does diet strictness level during weekends and holiday periods influence 1-year follow-up weight loss maintenance? Evidence from the Portuguese Weight Control Registry

**DOI:** 10.1186/s12937-019-0430-x

**Published:** 2019-01-11

**Authors:** Rui Jorge, Inês Santos, Vitor Hugo Teixeira, Pedro Jorge Teixeira

**Affiliations:** 1 Self-Regulation in Physical Activity, Nutrition and Obesity Research Group (PANO-SR), Interdisciplinary Center for the Study of Human Performance (CIPER), Faculty of Human Kinetics, University of Lisbon, Estrada da Costa, 1495-687 Cruz Quebrada, Portugal; 2 Centro de Investigação Interdisciplinar Egas Moniz (CiiEM), Instituto Universitário Egas Moniz, Quinta da Granja, 2829-511 Monte de Caparica, Portugal; 3 Faculty of Nutrition and Food Sciences (FCNA), University of Porto, Rua Dr. Roberto Frias, 4200-465 Porto, Portugal; 4 Research Center in Physical Activity, Health and Leisure (CIAFEL), Faculty of Sports, University of Porto, Rua Dr. Plácido Costa, 91, 4200-450 Porto, Portugal

**Keywords:** Weight loss maintenance, Dieting, Diet strictness, Weekends, Holidays

## Abstract

There is not much evidence about how diet strictness during weekends and holidays influence long-term weight loss maintenance. Our aim was to examine how dieting more or less strictly during weekends and holidays (vs. weekdays and non-holiday periods) influence weight loss maintenance.

Participants (*n* = 108) from the Portuguese Weight Control Registry indicated whether they had a more or less strict diet regimen during weekends compared to weekdays. A similar question about holiday and non-holiday period’ diet regimen was answered. Weight and height were measured at baseline and 1y follow-up. A 3% maximum weight variation defined participants as “non-regainers”.

General level on dieting strictness on weekends vs. weekdays (*r* = − 0.28, *p* < 0.01) and holidays vs. non-holidays (*r* = − 0.33, *p* < 0.001) predicted 1y weight change.

Participants who reported being less strict on weekends (OR = 0.34, 95% CI: 0.15–0.81) were more likely to be non-regainers when compared with the ones who reported being more strict on weekends. Non-significant results were found during holidays (OR = 0.47, 95% CI: 0.20–1.09).

Adopting a less strict diet regimen during weekends, when compared to weekdays, was a behavioral strategy associated with long-term weight management in our sample.

## Introduction

Long-term weight loss maintenance is a key challenge. Even though many individuals report trying to lose weight [1], just between 17 to 23% are able to maintain weight loss [2–5].

It is not clear why evidence-based behavioral interventions work more effectively for some individuals than for others. An approach for understanding the individual variability observed in those interventions is studying the lifestyle patterns and identifying the behavioral characteristics of those who have been successful at long-term weight management [6].

The US National Weight Control Registry (NWCR) has been providing continuous insight into the process of weight loss maintenance over the past two decades [7]. In the last decade, a Portuguese [8], a German [9], a Greek [10], and a Finnish [11] Weight Control Registry were created with the same goal – investigating characteristics associated with weight loss maintenance and weight regain - therefore enhancing evidence and knowledge on successful weight loss maintenance.

The Portuguese Weight Control Registry (PWCR) is an ongoing voluntary registry of adults who have been successful at losing at least 5 kg and have maintained that weight loss for at least 1 year. Particularly, it aims to study the social, psychological, physiological and behavioral characteristics of Portuguese successful weight loss maintainers and explore how those are associated with weight loss and maintenance [8, 12].

An example of behavioral strategies used by these successful weight loss maintainers for achieving higher success includes higher levels of physical activity, walking, weight self-monitoring and establishing specific goals [12].

There is not much evidence about how diet’ strictness during weekends and holidays influence long-term weight loss maintenance. On the one hand, a more flexible dietary pattern on weekends and holidays may reduce boredom, which can precipitate dieting lapses, and allow a more realistic journey from a long-term perspective. On the other hand, being more flexible may increase exposure to high-risk situations, creating more opportunity for loss of control [13]. Within the NWCR, Gorin et al showed that participants who maintained a more consistent diet during weekends and holidays had 1.5 times more chances to maintain the weight lost than participants who reported more oscillations in their diet during those periods [6]. To our knowledge, no other Weight Control Registry explored these features.

Therefore, this study aims to examine how dieting more or less strictly during weekends and holidays, comparing to weekdays and non-holiday periods, influence weight loss maintenance in a Portuguese sample of successful weight loss maintainers.

## Methods

### Subjects

From 388 participants that entered the PWCR, 226 completed baseline laboratory assessments and 108 (61.1% women) completed 1y follow-up laboratory assessments. Only those who completed laboratory assessments at both baseline and follow-up (*n* = 108) were included in this specific study.

Detailed information regarding the methodology of the PWCR can be found elsewhere [12]. Briefly, participants were recruited from the community at large through local and national media coverage and advertisements, the PWCR website and the PWCR Facebook page. To be eligible for enrolment in the PWCR, all participants needed to have Portuguese nationality, be aged between 18 and 65 years old, and have maintained at least 5 kg intentional weight loss for at least 1y, independently of their initial body weight. All the individuals who met the eligibility criteria were invited to perform all the assessments at the Exercise and Health Laboratory of the Faculty of Human Kinetics, University of Lisbon. Those who could not visit the Laboratory received by mail a (partial) battery of questionnaires.

### Assessments

Upon entering the PWCR, all participants answered a questionnaire with standard demographic information, weight history details, and specific weight loss and weight maintenance behavioral strategies. Diet strictness level was obtained through a subjective assessment of the examined subjects with the following questions: “During the weekend do you maintain the same diet regimen that you adopt during the week?” and “During holidays do you maintain the same diet regimen that you adopt during the rest of the year?”, answered on a 7-point scale, from 1 (more strict during the weekend/holidays) to 7 (less strict during the weekend/holidays). A derived variable was created - diet strictness score – by calculating the mean of the two variables (lower diet strictness score for those adopting a less strict diet regimen and higher diet strictness score for those adopting a stricter diet regimen). This questionnaire was also answered at 1-year follow-up assessment.

In the laboratory, body weight was measured twice, using an electronic scale calibrated on site and accurate to 0.1 kg (SECA, Hamburg, Germany). Height was measured with a balance-mounted stadiometer to the nearest 0.1 cm. All assessments occurred according to standard procedures [14] at both assessment moments. Based on these variables, body mass index (BMI) and the magnitude of weight change (from baseline to 1-year follow-up) were calculated. A 3% maximum weight variation was considered to classify participants as “non-regainers” [15].

### Statistics and data analysis

Statistical analyses were conducted using IBM Statistical Package for the Social Sciences (SPSS) version 25 for Microsoft Windows. Significance level was set at *p* < 0.05 for all tests. Descriptive results are expressed in terms of group means and standard deviation for continuous variables and relative frequencies for categorical variables. All variables were tested for normality of distribution using the Kolmogorov-Smirnov test, kurtosis and skewness values. Independent-sample t tests for continuous variables and Chi-square tests for categorical variables were used to compare differences between those dieting more strictly during weekends and holidays vs. those dieting less strictly during weekends and holidays. Both Pearson’s and Spearman’s correlations were conducted to examine associations between diet strictness levels (non-normally distributed) and 1y weight change. Since Pearson correlations are robust against deviations from normal distribution in moderately large samples [16] and the differences between Pearson and Spearman correlations’ coefficients were minimal, only the parametric results were reported. Odds-ratio tests were conducted to determine if there were different probabilities of weight regain for participants dieting more strictly during weekends and holidays vs. those dieting less strictly during weekends and holidays.

## Results

Participants were 40.3 ± 10.7 years, most had completed higher education (73.3%), weighed 73.0 ± 13.4 kg and had a BMI of 26.4 ± 10.6 kg/m^2^. Before entering the study, they lost, on average, 17.9 kg or 24.5% of initial body weight (men: 27.3%; women: 22.7%, *p* > 0.05), and maintained that weight loss for ≈28 months. The mean weight difference between the 1y follow-up assessment and the baseline assessment was 0.5 ± 4.0 kg (men: 1.2 kg ± 3.8 kg; women: − 0.6 kg ± 4.0 kg, *p* = 0.018).

**Fig. 1:**
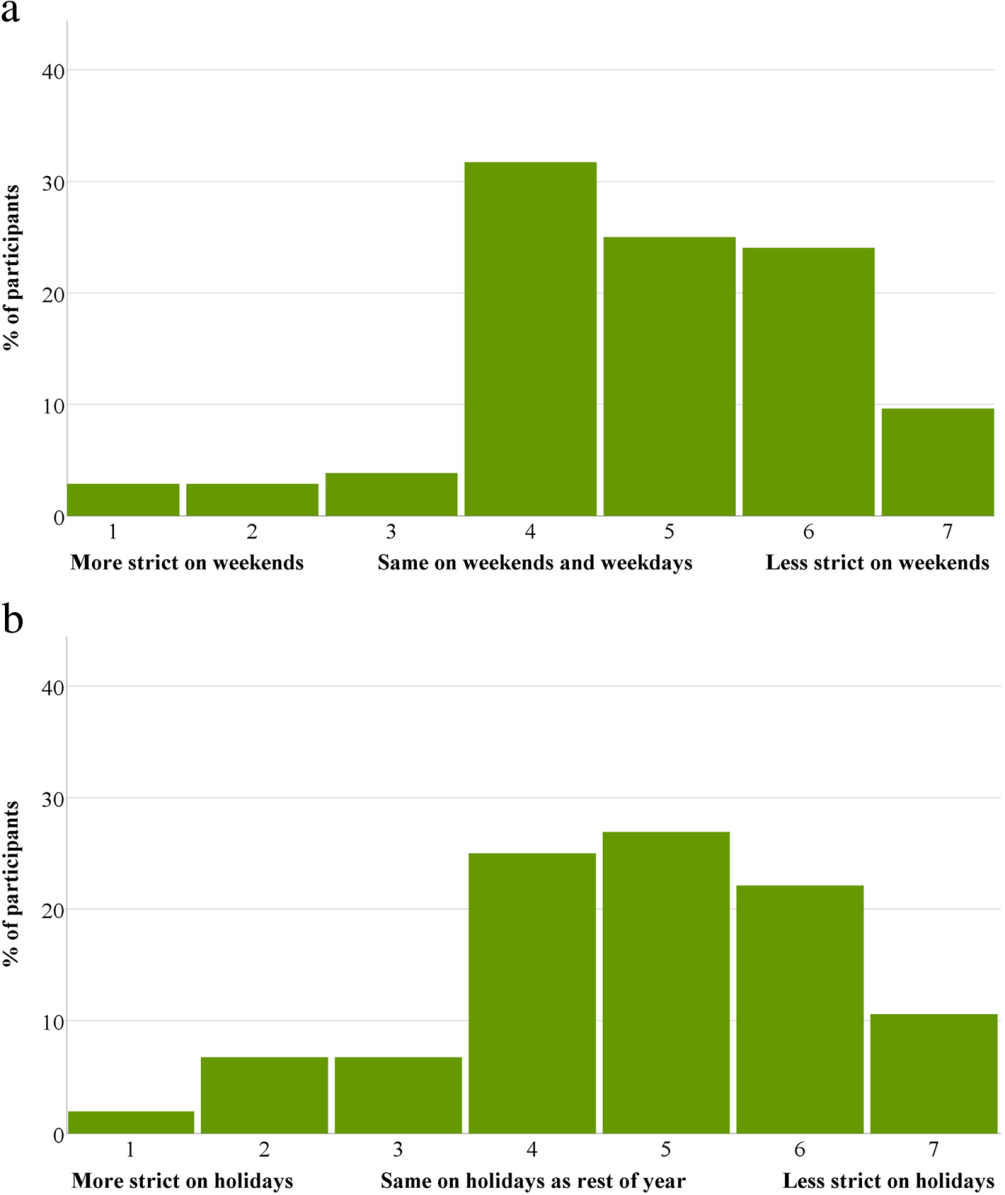
(a and b) Diet strictness levels in PWCR participants

The distributions of participants’ levels on the diet strictness scale questions are displayed in Fig. 1a and b.

About 9.6% of participants reported dieting more strictly on weekends, 31.7% reported maintaining the same diet and 58.7% reported dieting less strictly on weekends. Similarly, 15.4% reported dieting more strictly during holidays, 25.0% reported maintaining the same diet and 59.6% reported dieting less strictly during holidays. There were no statistically significant differences between the weekend vs. weekdays and the holidays vs. non-holiday periods levels (*p* > 0.05). There were also no statistically significant differences between those dieting more strictly during weekends and holiday periods compared to those dieting less strictly during weekends and holiday periods in terms of age, initial weight loss, and duration of weight loss maintenance (*p* > 0.05).

Participants who reported being less strict on weekends had a − 0.65% weight change from baseline to 1y follow-up; participants who reported being as strict on weekends as in the weekdays gained 2.60% of their body weight from baseline to 1y follow-up; and participants who reported being more strict on weekends gained 4.96% of their body weight from baseline to 1y follow-up. In the holiday’s question, weight change results were − 0.40%, 1.43% and 5.22%, respectively.

General level on diet strictness for weekends vs. weekdays and holidays vs. non-holiday periods predicted 1y weight change (*r* = − 0.28, *p* < 0.01 and *r* = − 0.33, *p* < 0.001, respectively). Participants who reported being less strict on weekends were more likely to be non-regainers when compared to the ones who reported being more strict during weekends (OR = 0.34, 95% CI: 0.15–0.81). Non-significant results were found for the holiday period (OR = 0.47, 95% CI: 0.20–1.09).

**Fig. 2:**
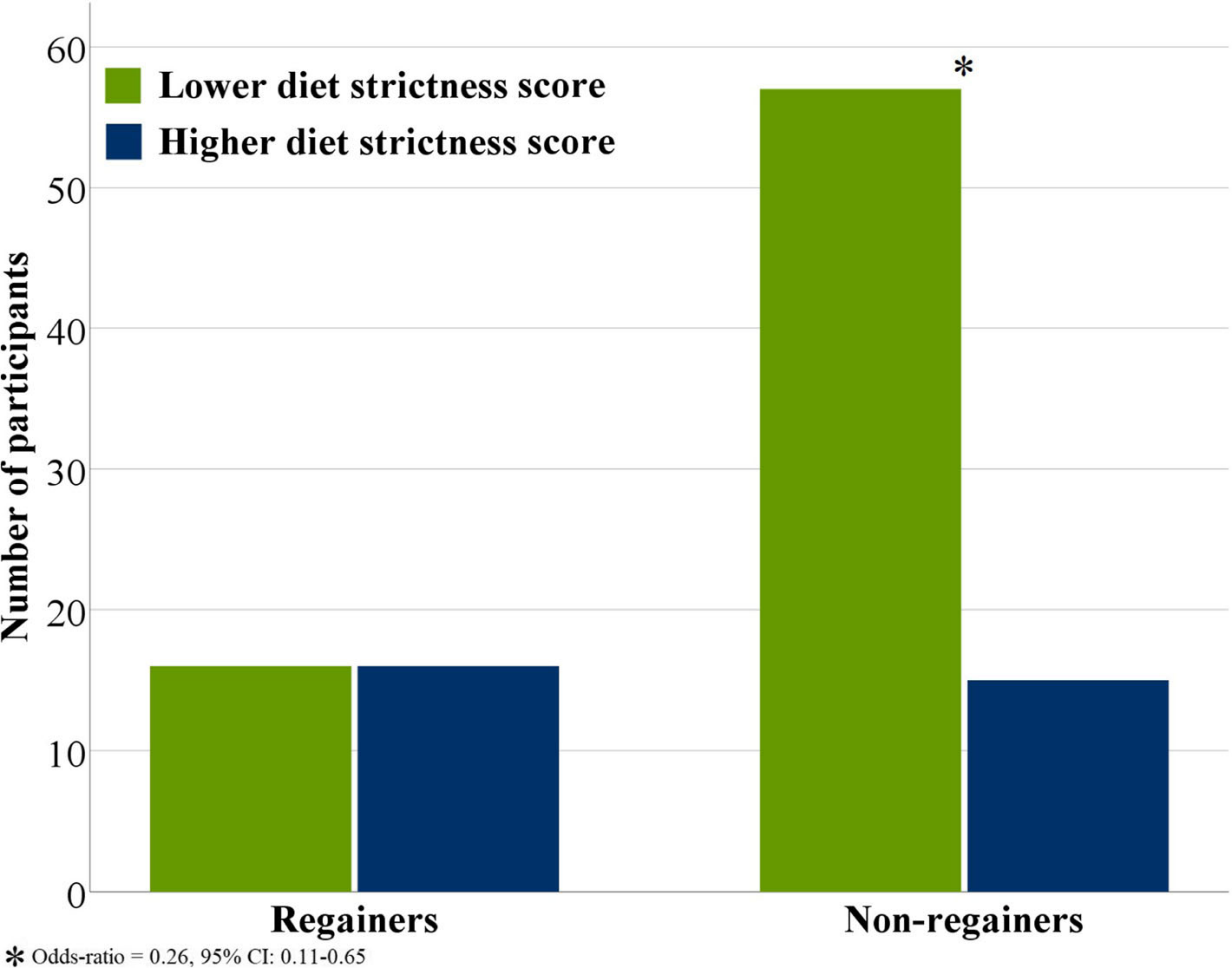
Clustered bar chart on Regainers and Non-regainers for lower and higher diet strictness score

Diet strictness score was inversely correlated with 1y weight change (*r* = − 0.34, *p* < 0.001): participants who were less strict on weekends and holidays were more likely to be non-regainers when compared to those who were stricter on weekends and holidays (OR = 0.26, 95% CI: 0.11–0.65) (Fig. 2).

## Discussion

This study sought to examine how diet strictness influence weight loss maintenance. Participants who reported dieting more strictly during weekends had statistically significant higher probability of regaining more than 3% of their weight in 1y, when compared to those reporting dieting less strictly during weekends. When diet strictness on weekends and diet strictness on holidays were computed into a mean score, similar statistically significant results emerged.

Predictors of successful long-term weight loss maintenance studied in the literature are often strategies that cannot be trained or taught to assist in weight control (e.g., lifetime weight cycling, maximum lifetime weight, magnitude of weight loss, duration of weight loss, disinhibition or depressive symptoms) [17]. Nevertheless, this brief report identifies a strategy that relies on behavioral processes and can be modifiable for long-term weight loss and maintenance, therefore providing additional clues for individuals attempting to control their weight.

These results confirm previous findings [6] and may be linked to a more rigid approach towards eating behavior. In fact, there were statistically significant differences between those dieting more strictly during weekends and holiday periods and those dieting less strictly during weekends and holiday periods in terms of rigid eating restraint levels (*p* < 0.05; data not shown). Weight loss maintainers often report experiencing higher burden and expressing effortful control to achieve weight loss maintenance than lifetime normal stable-weight individuals [18]. This higher perception of burden, rigid patterns, and the constant refrain from energy-dense foods, which can be more accessible or more “socially consumed” during weekends, can be deleterious in the long run, leading to vicious cycles of overeating and restriction, feelings of guilt, and weight regain.

Despite the convenience nature of the PWCR sample, which potentially makes it non-representative of the population of successful weight loss maintainers in Portugal, the PWCR provides a good setting for identifying critical factors for weight loss and maintenance and this study suggests that maintaining a consistent, more flexible diet across the entire week and year may prevent long-term weight regain. Nevertheless, the scarcity of literature available on the topic demands more research to determine whether instructing individuals to have a more flexible approach towards eating behavior on weekends and/or on holiday periods can improve their weight control success.

## Conclusions

Adopting a less strict diet regimen during weekends, when compared to weekdays, was a behavioral strategy associated with long-term weight management in our sample of previously successful weight loss maintainers. Advising a stricter dietary approach during the weekend, when compared to weekdays, can be counterproductive and should be avoided in those trying to maintain their weight loss.

## Abbreviation

BMI: Body mass index
NWCR: National Weight Control Registry
PWCR: Portuguese Weight Control Registry
